# Contemporary Fixed-Duration Treatment Options in the First-Line Setting of Chronic Lymphocytic Leukemia: Perspectives from a Publicly Funded Healthcare System

**DOI:** 10.3390/curroncol32100543

**Published:** 2025-09-28

**Authors:** Christopher Lemieux, Chai W. Phua, K. Sue Robinson, Carolyn Owen, Versha Banerji

**Affiliations:** 1 Division of Hematology and Medical Oncology, Centre Intégré de Cancérologie, CHU de Québec-Université Laval, 2250 Boul. Henri-Bourassa, Québec City, QC G1J 0J9, Canada; 2 Division of Hematology, Department of Medicine, Western University, 800 Commisioners Road East, London, ON N6A 5W9, Canada; 3 Division of Hematology, QEII Health Sciences Centre, Dalhousie University, 1247 South Park Street, Halifax, NS B3H 2Y9, Canada; 4 Division of Hematology and Hematological Malignancies, Arthur J.E. Child Comprehensive Cancer Centre, 3395 Hospital Drive NW, Calgary, AB T2N 5G2, Canada; 5 Department of Internal Medicine and Biochemistry and Maedical Genetics, Rady Faculty of Health Sciences, Max Rady College of Medicine, University of Manitoba, CancerCare Manitoba, 750 Bannatyne Avenue, Winnipeg, MB R3E 0T4, Canada

**Keywords:** chronic lymphocytic leukemia, fixed duration, continuous, venetoclax, obinutuzumab, ibrutinib, acalabrutinib

## Abstract

First-line options for chronic lymphocytic leukemia (CLL) are evolving, recently returning to a fixed-duration (FD) approach incorporating regimens such as venetoclax + obinutuzumab, ibrutinib + venetoclax, and soon acalabrutinib + venetoclax ± obinutuzumab. Five Canadian hematologists convened to share perspectives regarding the attributes of these options and considerations for clinically appropriate integration within Canada’s publicly funded healthcare system. The hematologists underscored the importance of shared decision-making with patients, family members, and caregivers involving careful consideration of disease profile and patient characteristics, preferences, and values. They indicated that although a role persists for continuous therapy with approved covalent Bruton’s tyrosine kinase inhibitors (typically in high-risk disease), newer FD regimens offer multiple benefits related to the treatment-free period, quality of life, safety, re-treatment, healthcare resource utilization, and costs. The hematologists highlighted the appeal of all-oral FD combinations given their convenience and impact on treatment equity, factors especially compelling given Canada’s vast geography and large segment of rural populations. In closing, they emphasized the quickly evolving therapeutic setting of CLL in the 1L and beyond, underscoring the need for ongoing patient involvement in decision-making to support optimal treatment selection based on patient goals and within the confines of provincial funding.

## Simple Summary

Contemporary drug combinations are or will soon be available for the first-line treatment of chronic lymphocytic leukemia that are provided to patients within a fixed duration. Hematologists from across Canada met to discuss the attributes of these options and share considerations for their use in clinical practice. The hematologists identified many benefits related to these therapies (e.g., time off treatment, improved patient quality of life and safety, reduced cost burden), though noted that a need likely remains for continuously administered options for certain patient subgroups. They stated that new all-oral fixed-duration combinations may be particularly appealing to Canadian patients and clinicians, flagging their associated convenience and treatment equity. The hematologists also emphasized the need for shared decision-making between patients, caregivers, and clinicians to ensure informed and optimal treatment selection that acknowledges patient characteristics and goals and drug availability within a publicly funded healthcare system.

## 1. Introduction

Therapeutic options for first-line (1L) treatment of chronic lymphocytic leukemia (CLL) have advanced substantially over the past decade. Previously, initial management consisted primarily of fixed-duration (FD) therapy with cancer center-delivered chemoimmunotherapy (CIT) combinations such as fludarabine-cyclophosphamide-rituximab (FCR), bendamustine-rituximab (BR), or chlorambucil-obinutuzumab (Clb+Obi), with selection determined by patient age, comorbidities, fitness, and renal function [1]. However, high-risk patients, particularly those with *TP53* gene abnormalities or unmutated immunoglobulin heavy chain variable region (IGHV) status, experienced suboptimal efficacy with these regimens [2]. Additionally, recognition of an increased risk of hematologic-associated secondary cancers with CIT (in particular with FCR, despite the possibility of functional cure in some patients) and the availability of newer treatment options for higher-risk CLL ultimately diminished their use [3]. The introduction of the covalent Bruton’s tyrosine kinase inhibitors (BTKi) ibrutinib [4] (first generation) and acalabrutinib [5] and zanubrutinib [6] (second generation), first for patients with relapsed/refractory CLL and later as 1L therapy, represented a significant shift in treatment practice from FD to continuous therapy and intravenous to oral administration. These options provide high efficacy across all molecular and patient profiles but require ongoing outpatient monitoring for adverse events and treatment adherence.

With the goals of further deepening molecular responses and potentially reducing treatment burden, new FD combination regimens have subsequently emerged for the front-line treatment of CLL. Initially, most of these regimens included intravenous administration of an anti-CD20 antibody (e.g., obinutuzumab); however, the introduction of double-oral combinations has offered hope in terms of ease of administration and improved treatment equity. Findings from recent pivotal studies of 1L FD regimens are summarized in Table 1 and Table 2. Combination of the apoptosis regulator B-cell leukemia/lymphoma 2 (BCL-2) inhibitor venetoclax with obinutuzumab (VenObi) was evaluated in the phase III trials CLL14 (patients with comorbidities) [7,8] and CLL13 (fit patients without *TP53* aberrations) [9,10], both of which showed superior progression-free survival (PFS) compared with ClbObi and FCR/BR, respectively. Soon thereafter, favorable PFS outcomes were also shown with the all-oral regimen ibrutinib + venetoclax (IbrVen) in the phase II CAPTIVATE FD trial of younger, fit patients (including high-risk patients with *TP53* aberrations) [11] and the subsequent phase III GLOW trial of patients aged ≥65 years or <65 with comorbidities (without *TP53* aberrations) [12,13]. Most recently, the combination of acalabrutinib with venetoclax ± obinutuzumab (AcalVen or AcalVenObi) showed superior PFS compared with FCR/BR in the phase III AMPLIFY trial, which included fit patients without *TP53* abnormalities [14]. Several other FD regimens are under investigation, including zanubrutinib in combination with the second-generation anti-BCL-2 therapy sonrotoclax [15].

In Canada, the full impact of integrating newer 1L FD options remains to be fully realized, in part given pending regulatory decisions (at the time of this writing) but also inter- and intra-provincial variations in drug access and reimbursement (including for oral versus intravenously administered therapies) within the country’s publicly funded healthcare system. Although public funding of cancer therapies ensures equal access to treatments, this principle only applies within individual provinces: after Health Canada approval, each province determines its own criteria for drug access, often leading to differential availability between provinces. Additionally, the speed of review and funding approval varies province-to-province, causing access to be delayed in some regions relative to others. Rarely, a province may opt not to fund an agent due to budgetary concerns, resulting in disparities across the country. Canada’s vast geography albeit relatively small patient population (41.5 million individuals in ~8.8 million km^2^ in 2025 [16,17]) further complicates drug access, treatment patterns, resource availability, and overall equity of options for patients.

As of June 2025, only two of the aforementioned FD regimens, VenObi and IbrVen, were approved by Health Canada for 1L use in CLL [18,19]. Although VenObi had been funded across the country for several years, a duration of time allowing for meaningful clinician experience, IbrVen was only very recently funded in a few provinces; in others, access remained dependent on private insurance plans or compassionate access programs via industry sponsors. Regulatory approval of the other all-oral combination AcalVen as well as AcalVenObi was expected within the next few months. In general, all of these novel FD options were anticipated to offer multiple benefits to patients, clinicians, and healthcare systems by limiting the duration of therapy and ameliorating long-term toxicity compared with continuous use of covalent BTKi.

Given the impending availability of multiple FD regimens, the varying characteristics of these combinations, and nuances related to the Canadian healthcare setting, the goal of this manuscript was to understand and share the perspectives of Canadian hematologists regarding the attributes and integration of these therapies, both compared with more conventional continuous BTKi therapy and across FD options.

**Table 1:** Study design information and key efficacy outcomes in pivotal trials of novel 1L FD regimens in CLL.

Trial Name Design Reference(s)	Treatment Arms	Patient Population	Key Efficacy Outcomes			
			ORR	Survival		
				Median F/U	PFS	OS
CLL14 Phase III Al-Sawaf et al. Lancet Oncol. 2020 [7]; Fischer et al. N Engl J Med. 2019 [8]; Al-Sawaf et al. Blood. 2024 [20]	VenObi vs. CIbObi	Patients with CIRS > 6 or CrCl < 70 mL/min N = 432	VenObi: 84.7% CIbObi: 71.3%	76.4 mo	Overall Median: 76.2 vs. 36.4 mo HR 0.40, 95% CI 0.31–0.52; *p* < 0.0001 del(17p)/*TP53*m Median: 51.9 vs. 20.8 mo HR 0.56, 95% CI, 0.30–1.06 mIGHV Median: NR vs. 62.2 mo HR N/A uIGHV Median: 64.8 vs. 26.9 mo HR 0.30, 95% CI, 0.22–0.42	Median NR 6-yr rate: 78.7% vs. 69.2% HR 0.69, 95% CI 0.48–1.01; *p* = 0.052 High-risk groups: N/A
CLL13 Phase III Eichhorst et al. N Engl J Med. 2023 [9]; Fürstenau M et al. Lancet Oncol. 2024 [10]	VenObi, VenR, IbrVenObi vs. FCR/BR	Fit patients (CIRS ≤ 6, CrCl ≥ 70 mL/min) without *TP53* aberrations N = 926	VenObi: 96.1% FCR: 80.8%	50.7 mo	VenObi vs. FCR Overall Median: NR 4-yr rate: 81.8% vs. 62.0% HR 0.47, 97.5% CI 0.32–0.69; *p* < 0.0001 mIGHV HR 0.45, 95% CI 0.20–1.05; *p* = 0.0063 uIGHV HR 0.45, 95% CI 0.31–0.66; *p* < 0.0001	VenObi vs. FCR Median NR 4-yr rate: 95.1% vs. 93.5% HR N/A High-risk groups: N/A
CAPTIVATE Phase II, single-arm (FD cohort) Tam et al. Blood. 2022 [11]; Wierda et al. JCO 2024 [21]	IbrVen	Fit patients aged ≤ 70 years N = 159	96%	61.2 mo	Medians NR; 5-yr rates: Overall: 67% del(17p)/*TP53*m: 41% Complex karyotype: 57% mIGHV: 85% uIGHV: 68% del(11q): 64%	Median NR 5-yr rate: 96% High-risk groups: N/A
GLOW Phase III Kater et al. NEJM Evid. 2022 [12]; Niemann et al. Lancet Oncol. 2023 [13]	IbrVen vs. ClbObi	Age ≥ 65 years or < 65 with CIRS ≥ 6 or CrCl < 70 mL/min; no *TP53* aberrations N = 211	IbrVen: 86.8% ClbObi: 84.8%	46 mo	Overall Median: NR vs. 21.7 mo 42-mo rate: 74.6% vs. 24.8% HR 0.214, 95% CI 0.138–0.334; *p* < 0.0001 mIGHV 42-mo rate: 90.0% vs. 43.1% uIGHV 42-mo rate: 69.8% vs. 15.0%	Median NR 42-mo rate: 87.5% vs. 77.6% HR 0.487, 95% CI 0.262–0.907; *p* = 0.021
AMPLIFY Phase III Brown et al. N Eng J Med. 2025 [14]	AcalVen, AcalVenObi vs. FCR/BR	Fit patients without *TP53* aberrations N = 867	AcalVen: 92.8% AcalVenObi: 92.7% FCR/BR: 75.2%	40.8 mo	AcalVen vs. FCR/BR Overall Median: NR vs. 47.6 mo 36-mo rate: 76.5% vs. 66.5% HR 0.65, 95% CI 0.49–0.87; *p* = 0.004 mIGHV Median: NR vs. NR 36-mo rate: 86.0% vs. 79.9% HR 0.67, 95% CI 0.39–1.14 uIGHV Median: 51.5 vs. 43.3 mo 36-mo rate: 68.9% vs. 56.8% HR 0.69, 95% CI 0.49–0.97	AcalVen vs. FCR/BR Median: 57.8 mo vs. NR 36-mo rate: 94.1% vs. 85.9%; HR 0.33, 95% CI 0.18–0.56; *p* < 0.001 ^a^
					AcalVenObi vs. FCR/BR Overall Median: NR vs. 47.6 mo 36-mo rate: 83.1% vs. 66.5% HR N/A mIGHV Median: NR vs. NR 36-mo rate: 83.6% vs. 79.9% HR 0.58, 95% CI 0.32–1.02 uIGHV Median: NR vs. 43.3 mo 36-mo rate: 82.8% vs. 56.8% HR N/A	AcalVenObi vs. FCR/BR Median: NR vs. NR 36-mo rate: 87.7% vs. 85.9% HR 0.76, 95% CI 0.49–1.18; *p* = NS

Note: Cross-trial comparisons should be conducted with substantial caution given differences in study designs, patient populations, endpoint definitions, and follow-up durations. Efficacy outcomes reflect the most recent data available at the time of manuscript publication. ^a^ Nominal value. 1L, first line; Acal, acalabrutinib; B, bendamustine; C, cyclophosphamide; CI, confidence interval; CIRS, Cumulative Illness Rating Scale; Clb, chlorambucil; CLL, chronic lymphocytic leukemia; F, fludarabine; FD, fixed duration; F/U, follow-up; HR, hazard ratio; Ibr, ibrutinib; mIGHV, mutated immunoglobulin heavy chain variable region; mo, month(s); N/A, not available; NR, not reached; NS, not significant; Obi, obinutuzumab; OS, overall survival; R, rituximab; PFS, progression-free survival; uIGHV, unmutated immunoglobulin heavy chain variable region; Ven, venetoclax; yr, year.

**Table 2:** Selected safety outcomes in pivotal clinical trials of novel 1L FD regimens in CLL.

Trial Name Comparators	All AEs n (%)		Atrial Fibrillation n (%)		Hypertension n (%)		Neutropenia n (%)		Infection n (%)		Thrombocytopenia n (%)		Hemorrhage (Major) n (%)		Diarrhea n (%)		TLS n (%)	
	Any Grade	Grade ≥3	Any Grade	Grade ≥3	Any Grade	Grade ≥3	Any Grade	Grade ≥3	Any Grade	Grade ≥3	Any Grade	Grade ≥3	Any Grade	Grade ≥3	Any Grade	Grade ≥3	Any Grade	Grade ≥3
CLL14 [8] ^a^																		
VenObi	200 (94.3)	167 (78.8)	N/A	N/A	N/A	N/A	122 (57.5)	112 (52.8)	N/A	37 (17.5)	51 (24.1)	29 (13.7)	N/A	N/A	59 (27.8)	9 (4.2)	3 (1.4) ^b^	N/A
ClbObi	213 (99.5)	164 (76.6)	N/A	N/A	N/A	N/A	122 (57.0)	103 (48.1)	N/A	32 (15.0)	50 (23.4)	32 (15.0)	N/A	N/A	32 (15.0)	1 (0.5)	5 (2.3) ^b^	N/A
CLL13 [9] ^a,c^																		
VenObi	214 (93.9)	183 (80.3)	2 (0.9)	0 (0.0)	21 (9.3)	4 (1.8)	111 (48.7)	103 (45.2)	151 (66.3)	30 (13.2)	40 (17.5)	34 (14.9)	N/A	N/A	75 (32.9)	4 (1.8)	16 (7.0) ^d^	11 (4.8) ^d^
FCR/BR	204 (94.4)	166 (76.8)	4 (1.9)	1 (0.5)	6 (2.8)	3 (1.4)	105 (48.5)	98 (45.3)	129 (59.7)	40 (18.5)	32 (14.9)	18 (8.4)	N/A	N/A	27 (12.5)	1 (0.5)	3 (1.4) ^d^	3 (1.4) ^d^
CAPTIVATE [11]																		
(FD cohort) ^a^																		
IbrVen	N/A	N/A	7 (4)	2 (1)	25 (16)	9 (6)	66 (42)	52 (33)	106 (67)	13 (8)	94 (59) ^e^	20 (13) ^e^	3 (2)	2 (1)	99 (62)	5 (3)	0 (0)	0 (0)
GLOW [12] ^c^																		
IbrVen	N/A	80 (75.5)	15 (14.2)	7 (6.6)	14 (13.2)	8 (7.5)	44 (41.5) ^f^	37 (34.9) ^g^	N/A	18 (17.0) ^h^	12 (11.3)	6 (5.7)	N/A	N/A	54 (50.9)	11 (10.4)	N/A	0 (0.0)
ClbObi	N/A	73 (69.5)	2 (1.9)	0 (0.0)	5 (4.8)	2 (1.9)	61 (58.1) ^f^	52 (49.5) ^g^	N/A	12 (11.5) ^h^	28 (26.7)	21 (20.0)	N/A	N/A	13 (12.4)	1 (1.0)	N/A	6 (5.7)
AMPLIFY [14]																		
AcalVen	270 (92.8)	156 (53.6)	2 (0.7) ^i^	1 (0.3) ^i^	12 (4.1)	8 (2.7)	90 (30.9)	78 (26.8)	148 (50.9)	36 (12.4)	13 (4.5)	4 (1.4)	3 (1.0)	3 (1.0)	95 (32.6)	5 (1.7)	1 (0.3)	1 (0.3)
AcalVenObi	269 (94.7)	197 (69.4)	6 (2.1) ^i^	2 (0.7) ^i^	11 (3.9)	6 (2.1)	114 (40.1)	100 (35.2)	153 (53.9)	67 (23.6)	24 (8.5)	17 (6.0)	8 (2.8)	6 (2.1)	103 (36.3)	4 (1.4)	1 (0.4)	1 (0.4)
FCR/BR	236 (91.1)	157 (60.6)	2 (0.8) ^i^	2 (0.8) ^i^	7 (2.7)	2 (0.8)	99 (38.2)	84 (32.4)	82 (31.7)	26 (10.0)	33 (12.7)	22 (8.5)	2 (0.8)	1 (0.4)	28 (10.8)	1 (0.4)	8 (3.1)	8 (3.1)

Note: Cross-trial comparisons should be conducted with substantial caution given differences in study designs, patient populations, endpoint definitions, and follow-up durations. Results reflect safety outcomes evaluated immediately after completion of FD treatment. ^a^ Grade 5 AEs not included; ^b^ No event met the Howard criteria for clinical TLS (i.e., presence of specific electrolyte changes and clinical manifestations); ^c^ Some values calculated using published data; ^d^ Laboratory-confirmed TLS as defined by Cairo–Bishop criteria: defined as presence of ≥2 specific changes in uric acid, potassium, phosphorus, and corrected calcium within 3 days before or 7 days after treatment initiation/escalation; ^e^ Defined as “platelets decreased”; ^f^ Includes both neutropenia and neutrophil count decreased; ^g^ Includes neutrophil count decreased; ^h^ Includes multiple preferred terms; ^i^ Includes atrial flutter. 1L, first line; Acal, acalabrutinib; AE, adverse event; B, bendamustine; C, cyclophosphamide; CLL, chronic lymphocytic leukemia; Clb, chlorambucil; F, fludarabine; FD, fixed duration; Ibr, ibrutinib; N/A, not available; Obi, obinutuzumab; R, rituximab; TLS, tumor lysis syndrome; Ven, venetoclax.

## 2. Materials and Methods

A list of Canadian hematologists was compiled by a medical writer and AstraZeneca Canada. Based on clinical expertise in CLL (e.g., clinical trial participation) and geographic location (to support pan-Canadian representation), the medical writer invited six hematologists by email to share their opinions regarding 1L treatment options for CLL. Five of the six hematologists agreed to participate in manuscript development.

In collaboration with the senior authors, the medical writer developed a series of questions that centered on the following topics related to continuous and FD regimens: perceived/realized advantages and disadvantages, preferred patient demographics and disease profiles, and decision-making approaches. During a virtual meeting, all five hematologists provided feedback on the focus and phrasing of the questions, which were then refined by the medical writer. The final seven questions included the following:

1. What are your general thoughts on FD regimens in the 1L treatment of CLL?
2. In your opinion, how do FD regimens compare with continuous treatment options in terms of their potential or recognized (A) benefits and challenges for patients, and (B) impact on healthcare professionals (HCPs) and healthcare systems?
3. What key factors do you consider when choosing between an FD regimen and a continuous treatment approach?
4. What advantages and/or disadvantages do you consider to be associated with each of the following FD regimens: VenObi, IbrVen, AcalVen, AcalVenObi?
5. What concerns, if any, do you have with the safety profile of each of these FD regimens?
6. Which patient profiles or demographics would you consider most suitable for each FD regimen?
7. How do you involve patients in the decision-making process for selection between FD and continuous treatment options?

The hematologists individually responded to each question through a combination of written answers and one-on-one discussions with the medical writer. Follow-up queries were conducted by the medical writer as needed to obtain opinions on all pertinent considerations. The medical writer synthesized the responses with the goal of highlighting commonalities and differences among the hematologists. Each hematologist subsequently reviewed and refined the content to ensure their insights were captured fully and accurately.

## 3. Results & Discussion: Hematologist Insights

### 3.1. Comparison of Continuous BTKi and FD Regimens

The hematologists offered numerous insights regarding continuous versus FD options in the 1L treatment of CLL, giving consideration to patient, HCP, and healthcare system factors. These included the treatment-free period, efficacy and safety, opportunity for re-treatment, adherence, and cost and resource utilization. Throughout their responses, the hematologists consistently emphasized the importance of shared decision-making among patients, family members, and caregivers to promote holistic consideration of patient- and disease-related characteristics, preferences, values, and lifestyle, such that an optimal therapeutic approach can be identified. They also cautioned, however, that in the Canadian setting, treatment options can be limited by variations in funding status across provinces.

#### 3.1.1. Treatment-Free Period

All of the hematologists agreed that the treatment-free period associated with FD regimens represents a key advantage over continuous BTKi options, offering benefits to patients and cancer centers alike. Most believed that the post-treatment phase is likely associated with improved patient quality of life (QoL), which could arise from reduced follow-up healthcare encounters, lower rates of nuisance side effects, and the absence of the daily reminder of cancer that is imposed by indefinite drug therapy. One hematologist suggested that after completion of an FD regimen, the substantially reduced follow-up needs may better enable patients to return to “normal life”, which may include workforce participation, travel, and/or the ability to safely address other health concerns. Two others flagged a benefit for patients who become hospitalized or require surgery, as such situations are risky for individuals who neglect or forget to communicate their treatment with continuous BTKi therapy, potentially leaving them vulnerable to serious complications. Furthermore, multidisciplinary consultations and decision-making regarding treatment hold or discontinuation are often needed in such scenarios, increasing healthcare system burden.

#### 3.1.2. Efficacy, Re-Treatment, Safety, and Adherence

Two hematologists shared that although overall efficacy appears to be similar between 1L FD and continuous options (noting a current lack of comparative phase III trials), based on relatively short-term follow-up, the combination FD regimens are associated with deeper molecular responses that may appeal to and influence decision-making for some patients. One also flagged that FD treatment options have not been associated with the same degree of resistance-associated mutations as continuous BTKi therapy, an attractive characteristic given potential for re-treatment [22,23]. Several hematologists stated that FD regimens may also offer a reduction in cumulative toxicity compared with continuous BTKi therapy, although the rates of such toxicities are low overall. One noted that in follow-up studies of continuous treatments, many patients discontinued therapy because of side effects or a lack of desire to continue therapy long term [24,25,26]. They further remarked that there remains no ideal way to verify whether patients are taking their oral treatment. Other hematologists suggested that patient adherence to both drug therapy and monitoring protocols is likely higher with FD than continuous regimens, in part given the ramp-up requirements of venetoclax but also because a finish line is in sight. They stated that having a clear end date can motivate patients and foster a more hopeful outlook.

#### 3.1.3. Costs and Resource Utilization

While acknowledging that precise cost estimates are uncertain, several hematologists speculated that overall budget impact should be lower with FD than with continuous regimens. This related to greater certainty regarding the length of FD treatment and therefore more predictable drug costs, as well as reduced resources and follow-up needs. Nonetheless, resource utilization was flagged to potentially be more challenging, especially early in the course of FD therapy and particularly with use of VenObi. One hematologist explained that depending on the FD regimen selected, lab and clinic visit requirements and the associated travel burden can be substantially higher than those for continuous therapy in the first 12 months. Moreover, multiple hematologists underscored that pre-treatment counseling is more intensive for FD than continuous BTKi therapy. One hematologist stated, “After year one, continuous BTKi patients keep having to return to the cancer center for check-up and renewal; so, slowly over time, the burden of physician work might shift towards continuous BTKis, but this is probably not on balance until year three of therapy.” Others commented that continuous therapy means that patients are followed more often and longer, which can be problematic both in terms of clinic capacity and the long wait lists faced by some centers in seeing new or sick patients.

#### 3.1.4. Ongoing Role of Continuous BTKi in the First-Line Setting

Despite the numerous perceived benefits of FD regimens, all the hematologists indicated that continuous BTKi monotherapy is likely to remain an important part of the CLL armamentarium. Several stated that certain high-risk patients, such as those with *TP53*-aberrant disease (i.e., del[17p] or *TP53* mutation) without cardiovascular disease (CVD), may achieve superior outcomes with continuous regimens over the long term, making them the preferred choice. Notwithstanding, some clinicians felt that FD BTKi + anti-BCL2 combinations still reflect an effective option in this setting, and that patient- and disease-related factors must be considered on a case-by-case basis.

The hematologists also noted that for some patients, continuous therapy may be perceived as an easier and less overwhelming option, particularly when compared to the logistical considerations associated with administration of combination regimens that require frequent visits or monitoring, such as venetoclax and/or obinutuzumab, as further detailed below. Similarly, non-academic HCPs often prefer continuous BTKi therapy because of limited local resources and/or expertise in delivering more complex FD regimens. This situation is particularly relevant for rural and remote settings where venetoclax ramp-up procedures can be much more difficult to administer than at academic centers.

Some hematologists further suggested that the long-term strategy for a patient, such as whether they are anticipated to receive only one line of therapy given age and/or frailty, can steer decision-making towards a simple and easily modifiable continuous approach. Moreover, they noted that continuous therapy can provide psychological reassurance to patients who are anxious that stopping treatment might lead to early relapse. For such individuals, ongoing BTKi treatment may feel safer in terms of maintaining CLL suppression. One hematologist shared, “Patient willingness to be on FD or continuous therapy can trump disease-related factors. Are they comfortable with FD, can they do it, do they have supports in place to get through it? If the answer is ‘yes’, we should give FD; if not, we need to support them with the next best thing, which is continuous. It comes at a greater cost to the system, but still gives patients access to an excellent treatment option.” Another commented that they prefer FD over continuous, because they think it is what they would want if they were making this decision for themself—the ability to have time off treatment while well and without active cancer.

### 3.2. Selection Between Novel FD Regimens

Reflecting on the benefits and challenges associated with currently and imminently available FD options, the hematologists highlighted multiple considerations related to the impact of treatment protocols, mode of administration, and safety profiles on convenience, resource utilization, and patient suitability. One stated that although all regimens appear to be efficacious and relatively safe, nuances related to patient comorbidities, preferences, and geographic location strongly influence treatment selection.

#### 3.2.1. Venetoclax + Obinutuzumab

Of the two FD approaches approved in Canada at the time of feedback, VenObi was stated to generally remain preferred and was used most frequently by the hematologists. This was largely driven by long-term data for the regimen [10,20], as well as the adverse safety profile of IbrVen (especially related to the cardiovascular [CV] complications of ibrutinib) and prevailing restrictions related to its access and reimbursement. Nonetheless, each hematologist mentioned the hurdles associated with the early stages of VenObi therapy, the intensity and logistics of which can negatively impact not only patients but also HCPs and systems. More specifically, they flagged the regimen’s potential and often unpredictable toxicity in terms of tumor lysis syndrome (TLS) related to venetoclax and severe infusion-related reactions (IRRs; e.g., hypotension, bronchospasm, atrial fibrillation) related to obinutuzumab, as well as early infections, cytopenias (especially neutropenia), and diarrhea (see Table 2). The hematologists explained that although these events are infrequent and now mostly mitigated through use of pre-medication, dose adjustments, and close monitoring, they remain associated with a considerable patient burden and high resource utilization and costs arising from multiple clinic visits, high staffing needs, and frequent laboratory testing during the first several weeks of therapy. They stated that patients receiving VenObi also incur out-of-pocket costs and inconveniences related to transportation/travel, parking, and the corresponding time commitment, especially for obinutuzumab infusions. One hematologist noted that the prolonged infusion time associated with obinutuzumab may be particularly challenging for frailer patients, though another mentioned that this issue has improved with the introduction of a rapid administration protocol. Patients living in rural communities, far away from their cancer/infusion center and/or testing laboratory (a common occurrence given Canada’s geography), were recognized to be most negatively impacted by the requirements associated with VenObi administration. Given some of the challenges associated with the treatment protocol, the hematologists emphasized the need for careful education and counseling to ensure patients are fully informed regarding its requirements. Variations across centers were described in terms of the multidisciplinary systems available to provide such support (e.g., via pharmacists and nurses). At some centers, much of this responsibility still falls on the hematologist, adding considerable burden related to the clinic time required to explain the treatment protocol.

Despite some challenges, the hematologists shared that onboarding procedures for VenObi are now well established and that the regimen’s efficacy and FD use support continued prescribing, potentially until easier, all-oral regimens are more widely available. One individual stated that after the burdensome start, the regimen becomes quite simple (i.e., once-monthly infusion) and is typically very well tolerated. Another emphasized benefits related to a lack of drug-drug interactions, avoidance of the need to follow hypertension, and minimal concern regarding surgeries/trauma necessitating drug discontinuation. Other hematologists shared that the rapid response to VenObi can be reassuring for patients.

When considering an ideal patient profile for VenObi, the hematologists agreed that most patients of all ages are suitable for the regimen, especially those with good (i.e., IGHV-mutated) or even intermediate-risk profiles who can handle a busy first month of therapy and are expected to achieve prolonged remission. Several remarked that VenObi is appealing given its suitability for patients with a history of CVD or elevated CV or bleeding risks, which as discussed below, can be a concern with BTKi combination options. Still, the need for good proximity to the infusion clinic and ongoing family/caregiver support was emphasized, as well as the absence of significant renal impairment and the ability to tolerate fluids given their role in managing TLS, should it occur. Two hematologists flagged that use of VenObi ensures patients receive obinutuzumab, the “best antibody available”, given the therapy is not currently funded in Canada in the setting of relapsed CLL. Another stated that treatment sequencing is indeed a consideration, although limited evidence is currently available to inform an optimal approach.

#### 3.2.2. All-Oral Options

The hematologists agreed that compared with VenObi, the current and impending all-oral FD options IbrVen and AcalVen are appealing in terms of their superior convenience and treatment equity, potentially being amenable for use regardless of patient location. One commented that these new options are “easier and more accessible, and having local blood work and moving away from the hospital is a big advance.” Several hematologists explained that as the lead-in period with covalent BTKi therapy reduces disease burden, the risk of TLS during venetoclax treatment is considered low, although ramp-up requirements (e.g., monitoring, blood work) are not less than those associated with VenObi [27]. Moreover, some stated that avoidance of obinutuzumab minimizes IRRs and frees up chair space at already overwhelmed infusion centers, in turn reducing healthcare system burden. Still, as described below, the hematologists raised some safety concerns related to baseline CV risk, particularly for IbrVen. One flagged that the pill burden (i.e., seven pills/day) associated with the all-oral regimens could potentially be a deterrent for some patients, in addition to side effects such as diarrhea, rash, and neutropenia. This hematologist also underscored the lack of head-to-head data for the all-oral regimens versus VenObi: “I don’t know if one is better, but you lose out on obinutuzumab if you choose [all-oral], which makes me want to keep favoring VenObi.” In contrast, another stated, “For anyone I’m considering for VenObi, I would probably favor an oral-oral combination.”

##### Ibrutinib + Venetoclax

As highlighted above, the hematologists described several benefits of IbrVen, the first all-oral regimen approved for 1L treatment of CLL in Canada, especially in terms of a reduced need for in-clinic visits. Nonetheless, they unanimously voiced concerns about off-target effects, such as the clinically significant rates of CV-related morbidity and mortality observed in the GLOW and CAPTIVATE trials [11,12]. The hematologists noted that in these studies, IbrVen was associated with any-grade and grade ≥3 hypertension, cardiac arrhythmias, and sudden cardiac death, with elevated rates observed versus ClbObi among the elderly participants enrolled in GLOW [12]. Given these findings, most of the hematologists expressed hesitation related to offering IbrVen to older patients (who are more likely to have CVD and/or other comorbidities), as well as younger individuals presenting with high CV risk (e.g., myocardial infarction in previous year; cardiac insufficiency). Nonetheless, the hematologists considered the regimen potentially beneficial for certain individuals with IGHV-mutated disease and even suitable for some with *TP53* aberrations, once again taking into account baseline risk and patient preferences. One shared, “To me, the CV risk has to be pretty severe to completely ignore using a BTKi.”

##### Acalabrutinib + Venetoclax ± Obinutuzumab

Although not yet available in Canada at the time of this writing, the hematologists expressed high anticipation for the AcalVen regimen based on results of the AMPLIFY trial [14]. Such interest related both to the therapy’s all-oral administration and potentially lower risk of off-target effects, especially severe CV complications and mortality, based on cross-trial comparisons versus IbrVen. Even so, some hematologists maintained a reluctance to use the regimen among patients with significant CVD or CV risk factors. One stated they would likely provide AcalVen to IGHV-mutated patients who refuse VenObi but still desire FD therapy. Another noted that both younger and older patients could benefit and that the regimen might be prioritized for those with IGHV-unmutated disease. This individual speculated that AcalVen will ultimately be the preferred option for most patients, largely because of the lack of in-clinic/hospital infusions. Another hematologist stated, “IbrVen would be great for the youngest, fit population, but AcalVen will be something that could transcend age and comorbidities and also potentially be used for frailer patients.”

The hematologists also discussed results for AcalVenObi from the AMPLIFY trial [14] and its anticipated use should the triplet combination become available in Canada. One stated that the regimen has the most potential of the FD regimens in terms of maximal disease eradication. This hematologist and others noted, however, that such efficacy appears to come at a cost of increased infection and mortality, though this primarily relates to trial conduct during the COVID-19 pandemic. Two others stated there is no clear signal as to where the regimen should be used, though another suggested it might be considered for patients with high-risk disease (e.g., very high tumor burden) and flagged that the addition of obinutuzumab seems to benefit patients with IGHV-unmutated disease.

### 3.3. Considerations for Shared Decision-Making

When queried about their approaches to and considerations for shared decision-making with patients with CLL, the hematologists again underscored the importance of providing information and discussing all relevant clinical and patient-related factors. One explained that as each patient approaches treatment selection differently, it is critical to thoroughly describe all options and clearly understand patient values. This individual noted that discussions must set expectations regarding anticipated effectiveness, potential toxicity, administration requirements, lifestyle impact, and the uncertainties associated with continuous and FD options, as well as the differential burden corresponding with specific regimen types. They flagged that given a cure is not yet available for CLL, it remains important to disclose that no individual approach is necessarily “one and done” and that there exists a tradeoff between treatment intensity and duration. Patients must also understand that a switch between continuous regimens may eventually be required, or re-treatment or use of a new therapy for those who experience relapse after FD treatment. The need for physicians to be aware of promising new regimens coming down the pipeline was also emphasized, such that patients can be appropriately informed given the often-long-term duration of the disease. Another hematologist similarly underscored that although comorbidities and side effects influence options, there is power in patient-HCP partnership and finding common ground, and that a fundamental relationship exists between education, treatment adherence, and honest conversations about risk. They flagged that being forthcoming about risk is especially important in the early-line setting, given the road ahead may be long. This hematologist further noted that it can be helpful if patients understand the high cost of their treatment regimen and that they have paid their dues via their taxes to have therapy covered: they explained that when patients find out, they are often extremely grateful and more likely to commit to completing therapy. The hematologist also stated that to provide more equitable care, institutions and organizations must do a better job evaluating challenges related to patients’ mobility, transportation, and support systems, and continue developing resources to eliminate issues these as differentiating factors. Several hematologists described sharing literature and/or videos with patients and caregivers to help better inform decision-making. Most also commented that although all treatment options are discussed, some patients want a specific recommendation. One reflected that, “I probably present FD as a preference, because that’s my internal/subconscious bias. I need a reason to recommend continuous.”

## 4. Conclusions and Looking Forward

Discussions with five hematologists practicing within Canada’s publicly funded healthcare system revealed a multitude of pertinent considerations impacting selection between continuous and FD treatment options, as well as among specific FD regimen types, in the 1L setting of CLL. Key factors included patient comorbidities (and to a lesser but related extent, age), proximity to treatment and testing centers, and individual values, preferences, and lifestyle. For several hematologists, molecular status also played a meaningful role, as well as issues related to costs and shared resources. The hematologists indicated that continuous covalent BTKi monotherapy will remain an integral part of the CLL toolbox, and that there will still be cases outside the high-risk population where such therapy will be used based on patient preference and circumstance. Nonetheless, newer FD options represent a significant change in current practice, and discussions regarding their advantages are warranted for both patients and healthcare systems. In particular, all-oral FD regimens that offer more favorable safety profiles and equitable access to treatment—characteristics that may be particularly compelling to more rurally located patient populations in both Canada and worldwide—are expected to trigger a paradigm shift and may in time become the new standard of care.

In finalizing their thoughts, the hematologists expressed hope related to the outlook of CLL in the 1L setting and beyond. They flagged the importance of readouts from phase III studies such as CLL17 (NCT04608318), which is comparing the efficacy of 1L continuous covalent BTKi monotherapy versus FD options, as well as MAJIC (NCT05057494), which is investigating the optimal duration of FD therapy based on clinical response and minimal residual disease (MRD) status. The hematologists additionally voiced interest in broader evidence for effective drug sequencing, rates of resistance mutation acquisition (or lack thereof) and implications for re-treatment, and the potential to improve clinical and financial toxicity through use of MRD-directed therapy. They shared anticipation for novel targeted drug therapies, particularly the new class of BTKi degraders, as well as the non-covalent BTKi pirtobrutinib and chimeric antigen receptor T-cell therapy, with investigations currently focused on later lines. One hematologist remarked, “Advancements are expected to keep coming; even if a patient is on an FD option and does not relapse for 5 to 10 years, the landscape will be entirely different once again.” Several of the hematologists reiterated that regardless of the number and types of therapies that become available, education of patients and consideration of their needs and wishes remain essential to optimizing the treatment selection process.

## Data Availability

No new data were created or analyzed in this study. Data sharing is not applicable to this article.
